# Identification of Ganglioside GM3 Molecular Species in Human Serum Associated with Risk Factors of Metabolic Syndrome

**DOI:** 10.1371/journal.pone.0129645

**Published:** 2015-06-23

**Authors:** Lucas Veillon, Shinji Go, Wakana Matsuyama, Akemi Suzuki, Mika Nagasaki, Yutaka Yatomi, Jin-ichi Inokuchi

**Affiliations:** 1 Division of Glycopathology, Institute of Molecular Biomembrane and Glycobiology, Tohoku Pharmaceutical University, Sendai, Japan; 2 Institute of Glycoscience, Tokai University, Kanagawa, Japan; 3 Department of Cardiovascular Medicine, Graduate School of Medicine, The University of Tokyo, Tokyo, Japan; 4 Department of Clinical Laboratory Medicine, Graduate School of Medicine, The University of Tokyo, Tokyo, Japan; University of Geneva, SWITZERLAND

## Abstract

Serum GM3 molecular species were quantified in 125 Japanese residents using tandem mass spectrometry multiple reaction monitoring. Individuals were categorized by the presence or absence of metabolic disease risk factors including visceral fat accumulation, hyperglycemia and dyslipidemia. A total of 23 GM3 molecular species were measured, of these, eight were found to be significantly elevated in individuals with visceral fat accumulation and metabolic disease, defined as the presence of hyperglycemia and dyslipidemia. All of the GM3 molecular species were composed of the sphingoid base sphingosine (d18:1 (Δ4)) and, interestingly, six of the eight elevated GM3 molecular species contained a hydroxylated ceramide moiety. The hydroxylated GM3 species were, in order of decreasing abundance, d18:1-h24:0 ≈ d18:1-h24:1 > d18:1-h22:0 » d18:1-h20:0 > d18:1-h21:0 > d18:1-h18:1. Univariate and multiple linear regression analyses were conducted using a number of clinical health variables associated with obesity, type 2 diabetes, metabolic disease, atherosclerosis and hypertension. GM3(d18:1-h24:1) was identified as the best candidate for metabolic screening, proving to be significantly correlated with intima-media thickness, used for the detection of atherosclerotic disease in humans, and a number of metabolic disease risk factors including autotaxin, LDL-c and homeostatic model assessment insulin resistance (HOMA-IR).

## Introduction

Type 2 diabetes is generally regarded as a lifestyle related disease, associated with a chronic caloric surplus, lack of exercise, obesity and high serum levels of LDL-c [1] and characterized by insulin resistance, hyperglycemia and relative insulin deficiency where the severity of each factor varies on a case by case basis [2].

In normal subjects, insulin triggers an array of biological activities that fall in to either mitogenic or metabolic changes. Upon insulin binding to the insulin receptor (IR), internal-tyrosine kinase activity is activated and phosphorylates IR. At this point, Shc may be phosphorylated, which in turn induces mitogenic signaling through activation of the Ras-MAPK pathway. Alternatively, the activated IR may stimulate metabolic signaling by recruiting and phosphorylating adaptor proteins including the insulin receptor substrate (IRS) protein. Phosphorylation of IRS leads to PI3-kinase activation which induces translocation of the GLUT-4 to the plasma membrane facilitating cellular glucose uptake [3]. It is known that the association of IR in caveolae microdomains (lipid rafts) in adipocytes is essential to execute complete insulin metabolic signaling [4].

Glycosphingolipids (GSLs) and their sialic acid-containing derivatives, gangliosides, are components of membrane lipids in which the lipid portion is embedded in the outer leaflet of the plasma membrane with the sugar chain extending to the extracellular space. The structural features of GSLs affect membrane fluidity and allow for microdomain formation, contributing to cell-cell interaction and receptor-mediated signal transduction [5]. We have previously shown that in cultured adipocytes in a state of tumor necrosis factor (TNF)α-induced insulin resistance, the depletion of GSLs by the inhibition of glucosylceramide synthase, which is the first step enzyme for the biosynthesis of all of GSLs, results in nearly complete recovery from the resistance of insulin receptor signaling [6]. A number of studies in animal models demonstrate that pharmacological inhibition of GSLs ameliorates insulin resistance and prevents some manifestations of metabolic syndrome [7–11]. Further, we have shown that expression of ganglioside GM3, which is the simplest ganglioside species synthesized by GM3 synthase (GM3S; also called SAT-I/ST3Gal-5), is increased in metabolic diseases [6,12]. *GM3S* gene expression and GM3 content are upregulated in visceral adipose tissue of obese model animals and serum GM3 levels are approximately 2-fold higher in obese patients with type 2 diabetes and/or dyslipidemia [12]. Moreover, *GM3S* deficient mice exhibit enhanced insulin signaling and less susceptibility to insulin resistance induced by a high fat diet [13,14]. These results imply that GM3 is responsible for insulin homeostasis.

We have postulated a working hypothesis “insulin resistance as a membrane microdomain disorder” [3,15,16] because of the fact that the abnormal increase of membrane GM3 in adipocytes induced by inflammatory cytokine TNFα resulted in the elimination of the IR from caveolae microdomains [17].

In 2008, we examined serum GM3 concentrations of patients with hyperlipidemia or type 2 diabetes, and found that serum GM3 concentrations were increased in type 2 diabetes with severe visceral fat accumulation (VFA) [12] and a patent was issued for the use of GM3 measurement as a method for detecting insulin-resistant diseases [18]. It is with this information in mind that we set out to determine which specific GM3 molecular species are implicated in metabolic disease. In this study, twenty three GM3 molecular species were quantified in individuals with or without VFA, hyperglycemia and dyslipidemia and, for the first time, the relationships between numerous metabolic disease risk factors and serum GM3 molecular species are reported. Our results indicate that levels of hydroxylated GM3 species in human serum correlate with a number of risk factors for metabolic and cardiovascular diseases (CVD).

## Research Design and Methods

### Study participants

One hundred and twenty five Japanese residents were recruited for the study. Twenty six subjects were healthy and without metabolic disease risk factors, 39 had VFA, 15 had VFA with hyperglycemia, 28 had VFA with accompanying dyslipidemia and 17 had VFA, hyperglycemia and dyslipidemia (metabolic disease). Data describing these individuals are found in Table 1. Hyperglycemia was defined as having a fasting blood glucose level greater than 126 mg/dL. Subjects were categorized as having dyslipidemia if any one of the following diagnostic values were above or below normal levels; triglycerides > 150 mg/dL, LDL-c > 140 mg/dL or HDL-c < 40 mg/dL. Serum lipids, glucose, insulin, renal, and liver enzymatic levels were measured in fresh blood samples obtained after more than 6 hr fasting. VFA was measured as previously reported [19]. All participants gave their written informed consent prior to their inclusion in the study. The experimental protocol was in agreement with international norms and approved by the ethics committee of The University of Tokyo.

**Table 1:** Subject Characteristics.

	Control subjects	Patients with VFA	Patients with VFA + hyperglycemia	Patients with VFA + dyslipidemia	Patients with VFA + hyperglycemia + dyslipidemia
n	26	39	15	28	17
Sex	8 men/18 women	29 men/10 women	13 men/2 women	25 men/3 women	16 men/1 women
Age (years)	51.8 ± 1.8	52.1 ± 1.5 ns	59.9 ± 2.1 **	50.1 ± 1.6 ns	54.4 ± 2.4 ns
BMI (kg/m^2^)	24.3 ± 0.2	27.5 ± 0.4 ****	27.3 ± 0.8 ****	28.1 ± 0.6 ****	28.8 ± 0.8 ****
Height (cm)	159.6 ± 1.5	168.2 ± 1.5 ***	167.4 ± 1.6 **	169.0 ± 1.5 ****	168.8 ± 1.8 ***
Weight (kg)	62.1 ± 1.1	78.0 ± 1.7 ****	76.5 ± 2.7 ****	80.3 ± 2.2 ****	82.3 ± 2.8 ****
Waist (cm)	83.3 ± 0.7	95.1 ± 1.0 ****	96.0 ± 2.2 ****	95.5 ± 1.5 ****	97.3 ± 1.7 ****
SBP (mmHg)	110.1 ± 1.9	113.7 ± 2.0 ns	121.0 ± 4.7 *	114.4 ± 2.5 ns	128.4 ± 3.1 ****
DBP (mmHg)	71.4 ± 1.1	72.2 ± 1.3 ns	74.8 ± 2.9 ns	75.4 ± 1.7 ns	82.6 ± 2.1 ****
TG neutral lipids (mg/dL)	85.1 ± 5.4	101.6 ± 3.8 *	96.9 ± 7.0 ns	266.5 ± 33.5 ****	275.7 ± 35.0 ****
HDL-c (mg/dL)	66.4 ± 2.5	54.2 ± 1.8 ***	57.0 ± 2.9 *	46.0 ± 1.8 ****	43.0 ± 2.3 ****
LDL-c (mg/dL)	121.4 ± 5.0	122.4 ± 4.5 ns	130.3 ± 5.8 ns	128.3 ± 7.5 ns	132.8 ± 8.9 ns
Fasting blood glucose (mg/dL)	92.2 ± 1.4	94.6 ± 1.2 ns	143.6 ± 8.4 ****	96.3 ± 1.2 *	127.2 ± 4.3 ****
Total cholesterol (mg/dL)	201.6 ± 5.3	192.6 ± 4.7 ns	204.5 ± 7.1 ns	208.6 ± 7.6 ns	210.7 ± 7.9 ns
Free fatty acids (μEq/L)	540.6 ± 53.8	470.3 ± 38.1 ns	444.5 ± 49.5 ns	527.7 ± 32.8 ns	410.8 ± 43.2 ns
Insulin (mIU/L)	5.0 ± 0.5	7.2 ± 0.7 *	9.5 ± 2.0 **	10.7 ± 1.7 **	12.8 ± 1.2 ****
HOMA-IR (mU/L)	1.1 ± 0.1	1.7 ± 0.2 *	3.4 ± 0.7 ***	2.5 ± 0.4 **	4.0 ± 0.4 ****
HOMA-β (%)	64.5 ± 6.8	93.0 ± 16.0 ns	46.4 ± 9.7 ns	121.3 ± 21.6 *	77.2 ± 8.2 ns

Means ± SE are presented. SBP, systolic blood pressure; DBP, diastolic blood pressure; TG, triglycerides; HOMA-IR and β, homeostatic model assessment insulin resistance and beta-cell function, respectively.

ns P > 0.05,

* P ≤ 0.05,

** P ≤ 0.01,

*** P ≤ 0.001,

**** P ≤ 0.0001

### Measurement of GM3

#### Total lipid extraction

One hundred, 1500 and 20 ng of GM3(d18:1-[^13^C]16:0), sphingomyelin(d18:1–17:0) and ceramide(d18:1–17:0) were added for internal standards to 50 μL of serum, respectively, the solution was lyophilized then dissolved in 5 mL of chloroform:methanol (1:1). The mixture was sonicated, incubated at 40°C for 1 h then centrifuged at 15,000 x g at 4°C for 30 min. The supernatant was retained and the pellet was subjected to the same extraction procedure. The first and second supernatants were combined and evaporated. Sphingolipid molecular species lipoform designations such as d18:1-h24:0 indicate a sphingosine (d = dihydroxy, 1 = number of double bonds) of 18 carbons and a 2-hydroxy (h) nervonic acid (24:1) residue within the ceramide portion.

#### Mass spectrometry

GM3, ceramide and sphingomyelin molecular species were quantified using HPLC coupled with electrospray ionization tandem mass spectrometry (MS/MS) in multiple reaction monitoring (MRM) negative ionization mode. The Thermo Fisher triple stage quadrupole (TSQ) Vantage AM (Waltham, MA) instrument was calibrated by directly infusing a mixture of GM3 species extracted from milk, all ion source parameters and ionization conditions were optimized to improve sensitivity. Total lipid extracts from 50 μL of serum were dissolved in 50 μL of methanol and 6 μL were injected onto a Thermo Fisher Accela 1250 HPLC pump (Waltham, MA) and separated using a Develosil carbon 30 column (C30-UG-3-1 x 50 mm, Nomura Co. Ltd, Japan). The gradient program employed started with 100% solvent A (20% H_2_O / 50% 2-propanol / 30% methanol containing 0.1% acetic acid and 0.1% ammonia) for 5 min then ramped to 100% solvent B (2% H_2_O / 50% 2-propanol / 48% methanol containing 0.1% acetic acid and 0.1% ammonia) over 30 min. One hundred percent solvent B was maintained for 4 min, then the solvent was returned to 100% solvent A over 1 min and held there for 10 min. The flow rate throughout the duration of the chromatographic run was 50 μL/min. A potential of -2500 V was applied between the ion source and the electrospray needle and nitrogen gas was used. The vaporizer temperature was 373°C, sheath gas pressure was 50 (arbitrary units), ion sweep gas pressure was 0 (arbitrary units), the auxiliary gas pressure was 15 (arbitrary units), the capillary temperature was 204°C, the declustering voltage was 0, the collision pressure was 1.0 mTor, the S-lens RF amplitudes were 276, 204 and 171 and the collision energies were 53, 29 and 46 eV for GM3, ceramide and sphingomyelin molecular species, respectively. A 0.01 s scan time was used, data were collected in profile mode and peak widths were Q1 full width at half maximum (FWHM) 0.7 and (FWHM) 0.7. MS/MS transitions are listed in S1 Table. The abundance of each molecular species was compared based on its relative percentage of the internal standards GM3(d18:1-[^13^C]16:0), ceramide(d18:1–17:0), and sphingomyelin(d18:1–17:0), of which 12, 0.4 and 30 ng were injected, respectively. Total GM3 values were calculated by taking the sum of the 23 molecular species detected. The following evaluation comes with a caveat: that all molecular species do not share identical ionization efficiencies. In this case, due to limited availability of pure molecular species standards, all species are assumed to have ionization efficiencies comparable to the internal standards used. Therefore, when evaluating the abundance of molecular species, detected amounts are compared that may not necessarily represent absolute amounts.

### Statistical analysis

Data are presented as mean ± SD. For all tests, *P* < 0.05 was deemed statistically significant. GM3 molecular species comparisons between groups were performed using the Mann-Whitney *U* test and Spearman’s rank correlation coefficients were calculated. For all other comparisons two-sample *t* tests were employed. All GM3 molecular species were subjected to multiple regression analyses where age, BMI, abdominal circumference, calcaneus stiffness, diastolic and systolic blood pressure, Brinkman index, ejection fraction, homeostatic model assessment insulin resistance (HOMA-IR), hemoglobin, platelets, aspartate aminotransferase (glutamic oxaloacetic transaminase) (AST (GOT)), alanine transaminase (glutamate-pyruvate transaminase) ((ALT (GPT)), γ-glutamyl transpeptidase (γ-GTP), triglycerides, creatinine, uric acid, HDL-c, C-reactive protein, LDL-c, free fatty acids, total adiponectin, autotaxin, sphingomyelin, phosphotidylcholine, lysophosphatidylcholine, and intima-media thickness (IMT) were investigated as explanatory variables (Table 2). All statistical analyses were conducted using GraphPad Prism 6.04 (GraphPad Software Inc., La Jolla, CA, USA) except multiple linear regression analyses when XLSTAT version 2014.4.02 (Addinsoft, New York, NY, USA) was utilized.

**Table 2:** Serum GM3 Molecular Species Multivariate Analysis.

	C24:0	C16:0	C24:1	C22:0	C23:0	C20:0	C22:1	C18:0	hC24:0	hC24:1	hC22:0	C18:1
**All**	LPC***	SM***	SM***		EF***	SM***	PLT***	BP****	LDL-c**	Age	ATX**	BP****
			LPC***		SM***	LPC***		SM***	LPC***	ATX**		SM***
					LPC***			LPC***		SM***		
**Male**		SM***	LPC***		LPC***	SM***	LPC***	BP****	LDL-c**	BP****		BP****
						LPC***		SM***		HOMA-IR**		SM***
								LPC***		SM***		
										LPC***		
**Female**				TG***	BP****	CRP***	LDL-c**	PLT***	AST**	Age	BMI*	
					Age				ALT**	HOMA-IR**	AST**	
									CR*****	Hb**	ALT**	
										AST**		
										ALT**		
										CRP***		

Clinical variables found to be significantly (*P* < 0.05) correlated with 12 most abundant GM3 molecular species by multiple regression analysis. LPC, lysophosphatidylcholine; SM, sphingomyelin; EF, ejection fraction; BP, blood pressure; ATX, autotaxin; CRP, C-reactive protein; HOMA-IR, homeostatic model assessment insulin resistance; AST, aspartate aminotransferase; ALT, alanine aminotransferase; TG, triglyceride; CR, Creatinine; PLT, platelets; Hb, hemoglobin.

Risk factors associated with

* obesity,

** metabolic disease,

*** atherosclerosis,

**** hypertension and

***** nephropathy.

## Results

### Subject characteristics

Individuals in the VFA, VFA with hyperglycemia, VFA with dyslipidemia and VFA with metabolic disease groups all had significantly higher BMI, height, weight, waist circumference, insulin levels, HOMA-IR scores and lower HDL-c values (Table 1). Individuals in the VFA, VFA with dyslipidemia and VFA with metabolic disease groups all had elevated triglyceride levels when compared to controls, however only the VFA with dyslipidemia and VFA with metabolic disease had values deemed pathological (>150 mg/dL) with averages of 266.5 ± 33.5 and 275.7 ± 35.0 mg/dL, respectively (Table 1). Fasting blood glucose was elevated to the point of a type 2 diabetes diagnosis (>126 mg/dL) in both the VFA with hyperglycemia and VFA with metabolic disease groups, average values of 143.6 ± 8.4 and 127.2 ± 4.3 mg/dL, respectively (Table 1).

### Serum GM3 molecular species levels in patients with risk factors for metabolic disease

**Fig 1:**
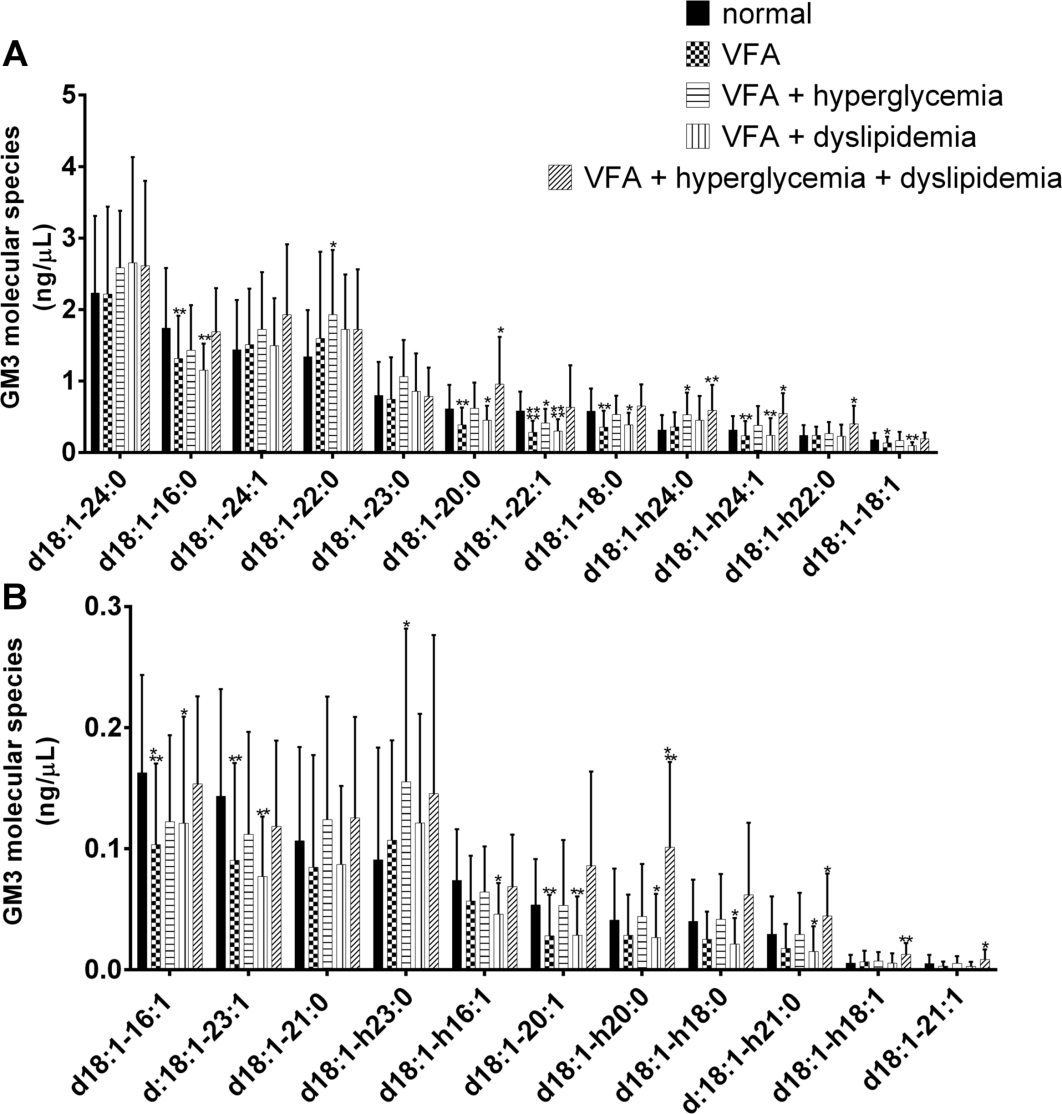
GM3 molecular species levels in human serum. Twelve most abundant (A) and eleven least abundant (B) GM3 molecular species detected in serum of patients with visceral fat accumulation (VFA) (n = 39), VFA with hyperglycemia (n = 15), VFA with dyslipidemia (n = 28) and VFA with both hyperglycemia and dyslipidemia (n = 17) compared with healthy lean control individuals (n = 26). Species were determined using LC-MS/MS MRM. Data are reported as means ± SD. * *P* ≤ 0.05, ** *P* ≤ 0.01, *** *P* ≤ 0.001, **** *P* ≤ 0.0001 metabolic risk factor groups vs. control; Mann-Whitney unpaired test.

**Fig 2:**
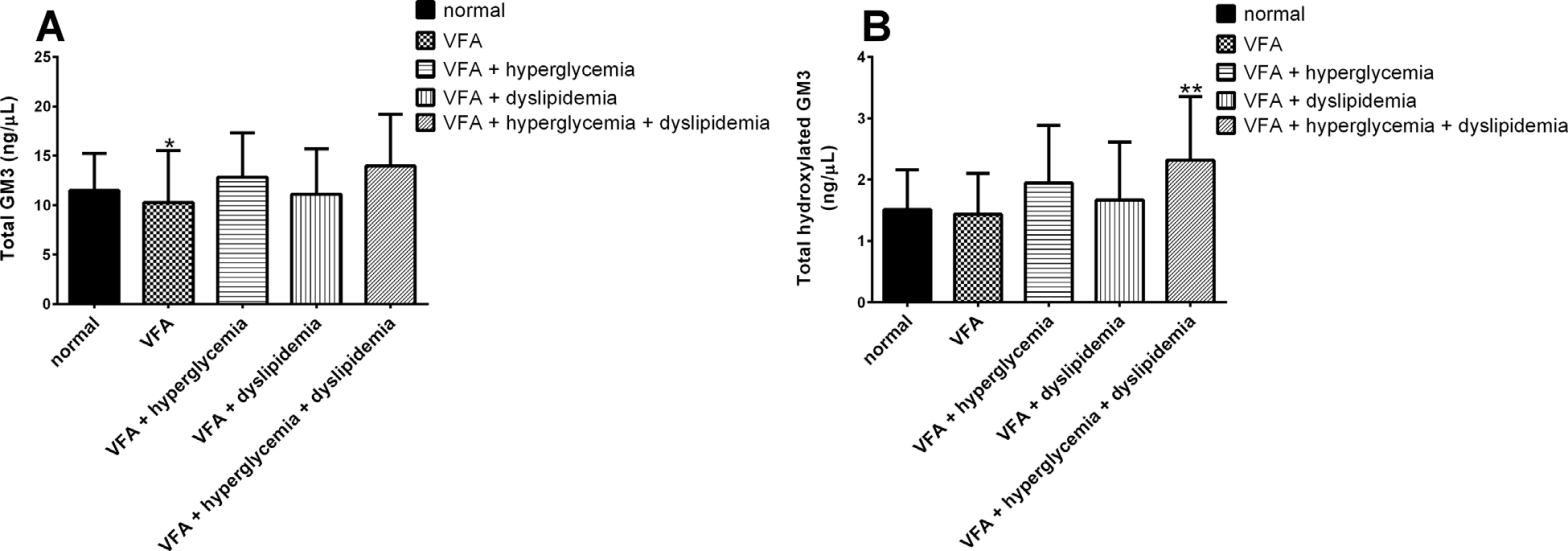
The sum of GM3 molecular species. Total GM3 (A) and total hydroxylated GM3 (B) detected in serum of patients with visceral fat accumulation (VFA) (n = 39), VFA with hyperglycemia (n = 15), VFA with dyslipidemia (n = 28) and VFA with both hyperglycemia and dyslipidemia (n = 17) compared with healthy lean control individuals (n = 26). Species were determined using LC-MS/MS MRM. Data are reported as means ± SD. * *P* ≤ 0.05, ** *P* ≤ 0.01, *** *P* ≤ 0.001, **** *P* ≤ 0.0001 metabolic risk factor groups vs. control; Mann-Whitney unpaired test.

The four most abundant molecular species of GM3 detected in control subjects were GM3(d18:1–24:0), GM3(d18:1–16:0), GM3(d18:1–24:1) and GM3(d18:1–22:0) (Fig 1A). Eight GM3 molecular species were elevated in a statistically significant fashion (*P* < 0.05) in the individuals of VFA with metabolic disease, in order of decreasing abundance they were GM3(d18:1–20:0) (control, 0.610 ± 0.336 ng/μL vs metabolic disease, 0.956 ± 0.662 ng/μL), GM3(d18:1-h24:0) (control, 0.317 ± 0.204 ng/μL vs metabolic disease, 0.588 ± 0.357 ng/μL), GM3(d18:1-h24:1) (control, 0.316 ± 0.192 ng/μL vs metabolic disease, 0.546 ± 0.283 ng/μL), GM3(d18:1-h22:0) (control, 0.238 ± 0.145 ng/μL vs metabolic disease, 0.400 ± 0.252 ng/μL), GM3(d18:1-h20:0) (control, 0.041 ± 0.042 ng/μL vs metabolic disease, 0.101 ± 0.070 ng/μL), GM3(d18:1-h21:0) (control, 0.029 ± 0.031 ng/μL vs metabolic disease, 0.044 ± 0.035 ng/μL), GM3(d18:1-h18:1) (control, 0.005 ± 0.007 ng/μL vs metabolic disease, 0.013 ± 0.009 ng/μL) and GM3(d18:1–21:1) (control, 0.005 ± 0.007 ng/μL vs metabolic disease, 0.009 ± 0.008 ng/μL), (Fig 1A and 1B). Additionally, the individuals of VFA with hyperglycemia exhibited elevated levels of GM3(d18:1–22:0) (control, 1.339 ± 0.651 ng/μL vs VFA with hyperglycemia, 1.927 ± 0.906 ng/μL), GM3(d18:1-h24:0) (control, 0.317 ± 0.204 ng/μL vs VFA with hyperglycemia, 0.527 ± 0.311 ng/μL) and GM3(d18:1-h23:0) (control, 0.091 ± 0.093 ng/μL vs VFA with hyperglycemia, 0.155 ± 0.126 ng/μL) (Fig 1A and 1B). Total GM3 values were elevated in the VFA with hyperglycemia and VFA with metabolic disease groups with average values of 12.814 ± 4.509 ng/μL and 13.972 ± 5.237 ng/μL respectively, compared with the control group’s average value of 11.468 ± 3.771 ng/μL (Fig 2A). However, this difference was not deemed statistically significant due to variation within the groups. Total hydroxylated GM3 species were elevated, and this increase observed in the VFA with metabolic disease group was statistically significant (control, 1.508 ± 0.651 ng/μL vs metabolic disease, 2.316 ± 1.041 ng/μL) with a *P* value of 0.0073 (Fig 2B).

### Correlation between GM3 molecular species and metabolic disease risk factors

**Fig 3:**
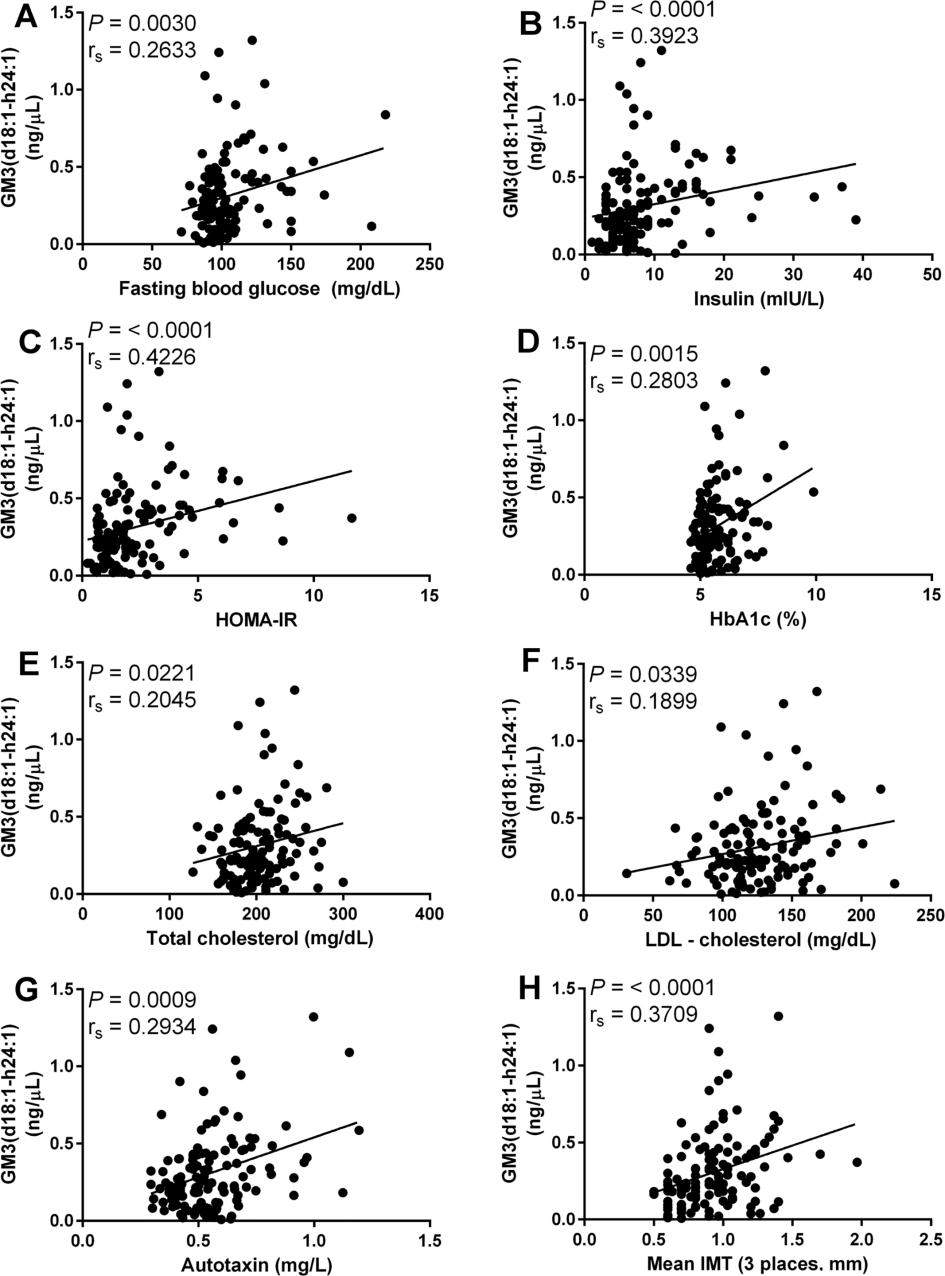
Association between metabolic disease risk factors and GM3(d18:1-h24:1). Correlation of GM3(d18:1-h24:1) with fasting blood glucose (A), insulin (B), HOMA-IR (C), HbA1c (D), total cholesterol (E), LDL-c (F), autotaxin (G) and mean IMT (H). Spearman’s rank correlation was used to access correlation between GM3(d18:1-h24:1) and metabolic disease risk factors. All correlations were deemed significant with *P* values below 0.05.

The results of multiple regression (Table 2) and univariate analyses indicated GM3(d18:1-h24:1) is significantly correlated with the largest number of metabolic disease risk factors. Spearman’s rank correlations were used to assess relationships between GM3 molecular species and clinical variables relevant to metabolic disease. Clinical variables associated with type 2 diabetes and atherosclerosis and correlated with GM3(d18:1-h24:1) include, but are not limited to, fasting blood glucose r_s_ = 0.2633 (mg/dL, Fig 3A), insulin r_s_ = 0.3923 (mIU/L, Fig 3B), HOMA-IR r_s_ = 0.4226 (Fig 3C), HbA1c r_s_ = 0.2803 (% glycated hemoglobin, Fig 3D), total cholesterol r_s_ = 0.2045 (mg/dL, Fig 3E), LDL-c r_s_ = 0.1899 (mg/dL, Fig 3F), autotaxin r_s_ = 0.2934 (mg/L, Fig 3G) and mean IMT r_s_ = 0.3709 (mm, Fig 3H).

**Fig 4:**
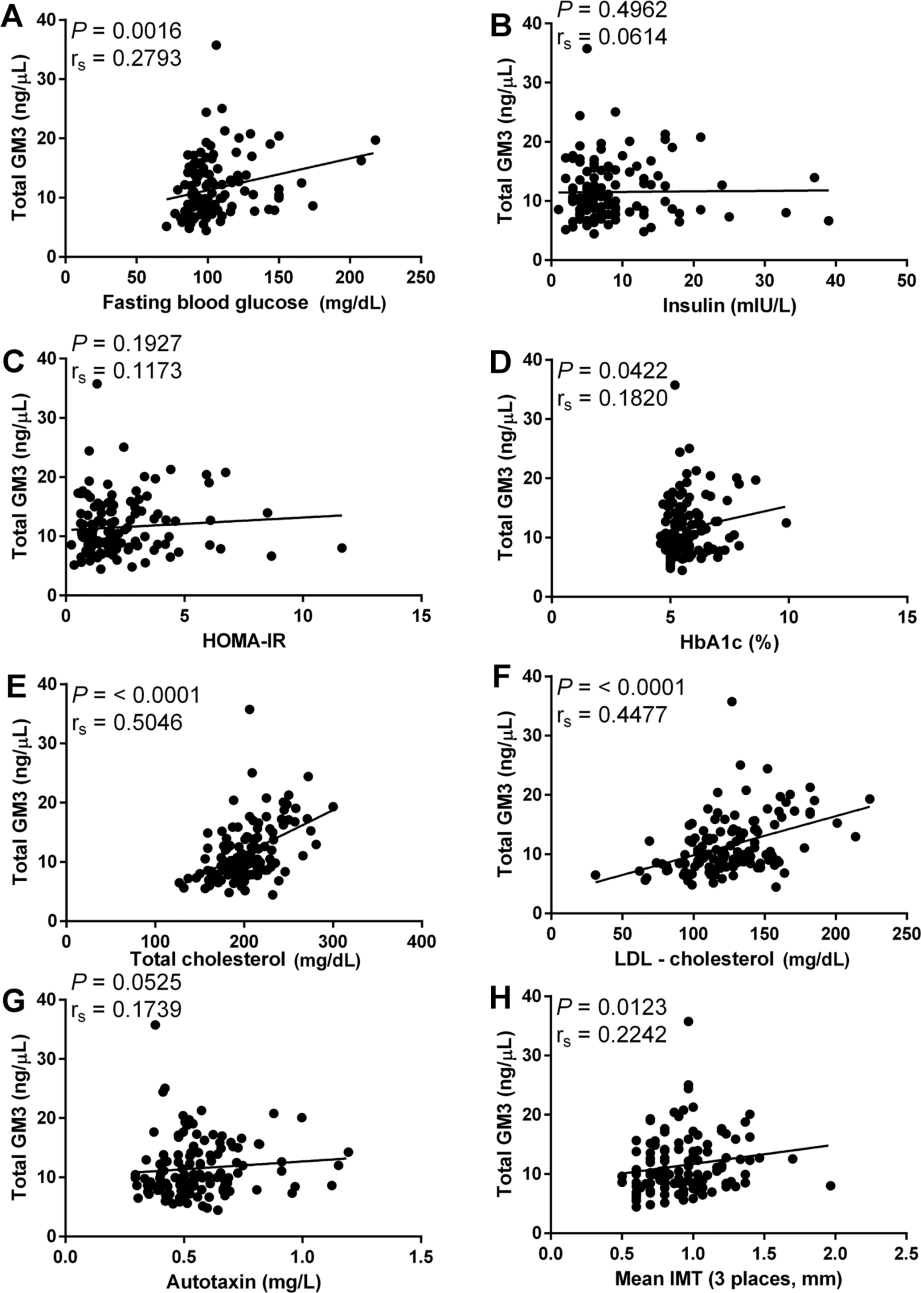
Association between metabolic disease risk factors and total GM3. Correlation of total GM3 with fasting blood glucose (A), insulin (B), HOMA-IR (C), HbA1c (D), total cholesterol (E), LDL-c (F), autotaxin (G) and mean IMT (H). Spearman’s rank correlation was used to access correlation between total GM3 and metabolic disease risk factors. Correlations of fasting blood glucose, HbA1c, total cholesterol, LDL-c and mean IMT with total GM3 were deemed significant with *P* values below 0.05.

**Fig 5:**
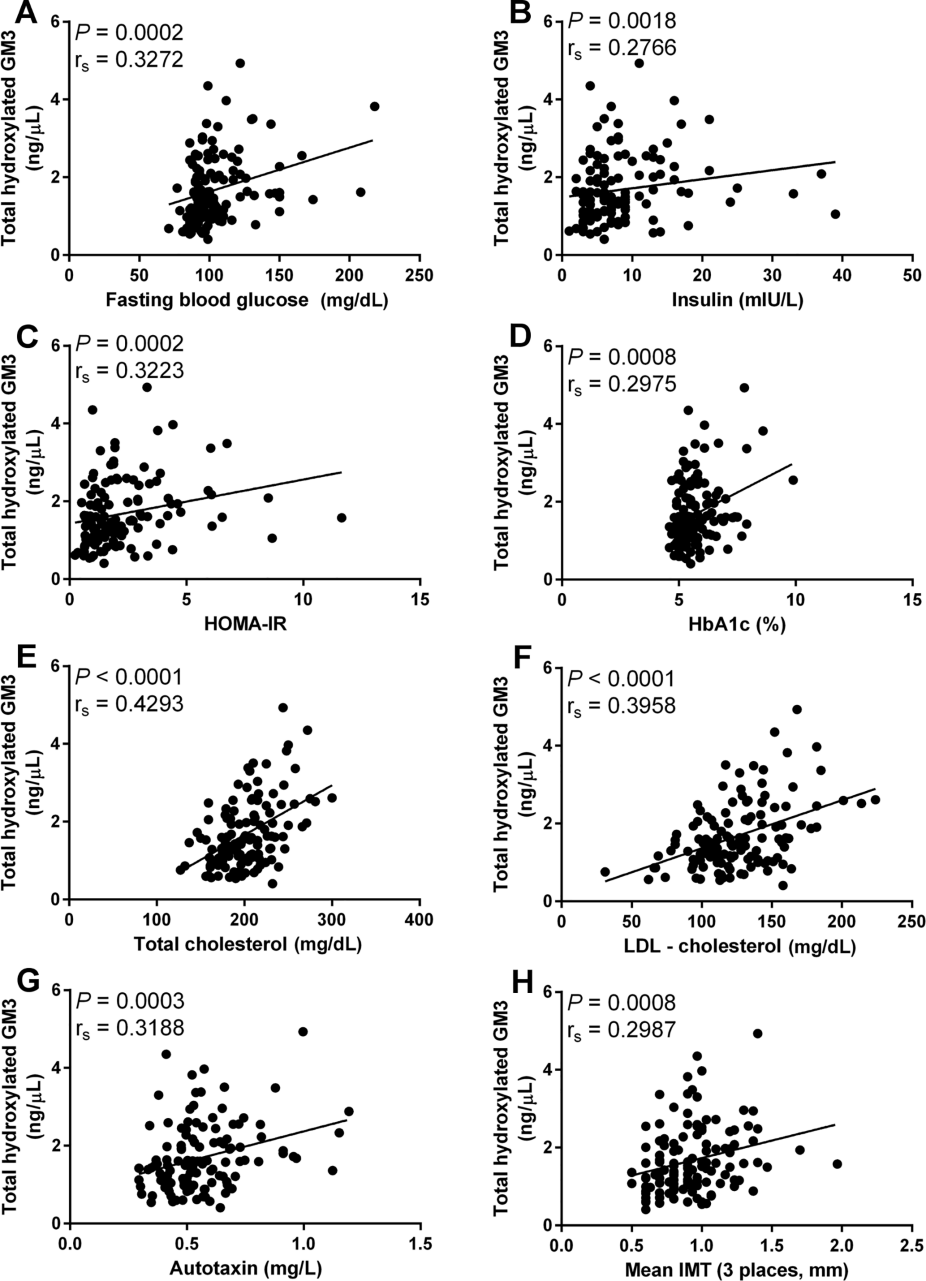
Association between metabolic disease risk factors and total hydroxylated GM3. Correlation of total hydroxylated GM3 molecular species with fasting blood glucose (A), insulin (B), HOMA-IR (C), HbA1c (D), total cholesterol (E), LDL-c (F), autotaxin (G) and mean IMT (H). Spearman’s rank correlation was used to access correlation between total hydroxylated GM3 molecular species and metabolic disease risk factors. All correlations were deemed significant with *P* values below 0.05.

In addition to GM3(d18:1-h24:1) it was found that total GM3 and total hydroxylated GM3 molecular species were correlated with risk factors for type 2 diabetes and CVD. Total GM3 was significant correlated with fasting blood glucose r_s_ = 0.2793 (mg/dL, Fig 4A), Hba1c r_s_ = 0.1820 (% glycated hemoglobin, Fig 4D), total cholesterol r_s_ = 0.5046 (mg/dL, Fig 4E), LDL-c r_s_ = 0.4477 (mg/dL, Fig 4F) and mean IMT r_s_ = 0.2242 (mm, Fig 4H). While total hydroxylated GM3 molecular species were significantly correlated with fasting blood glucose r_s_ = 0.3273 (mg/dL, Fig 5A), insulin r_s_ = 0.2766 (mIU/L, Fig 5B), HOMA-IR r_s_ = 0.3223 (Fig 5C), HbA1c r_s_ = 0.2975 (% glycated hemoglobin, Fig 5D), total cholesterol r_s_ = 0.4293 (mg/dL, Fig 5E), LDL-c r_s_ = 0.3958 (mg/dL, Fig 5F), autotaxin r_s_ = 0.3188 (mg/L, Fig 5G) and mean IMT r_s_ = 0.2987 (mm, Fig 5H).

### Relative abundance of GM3(d18:1-h24:1)

**Fig 6:**
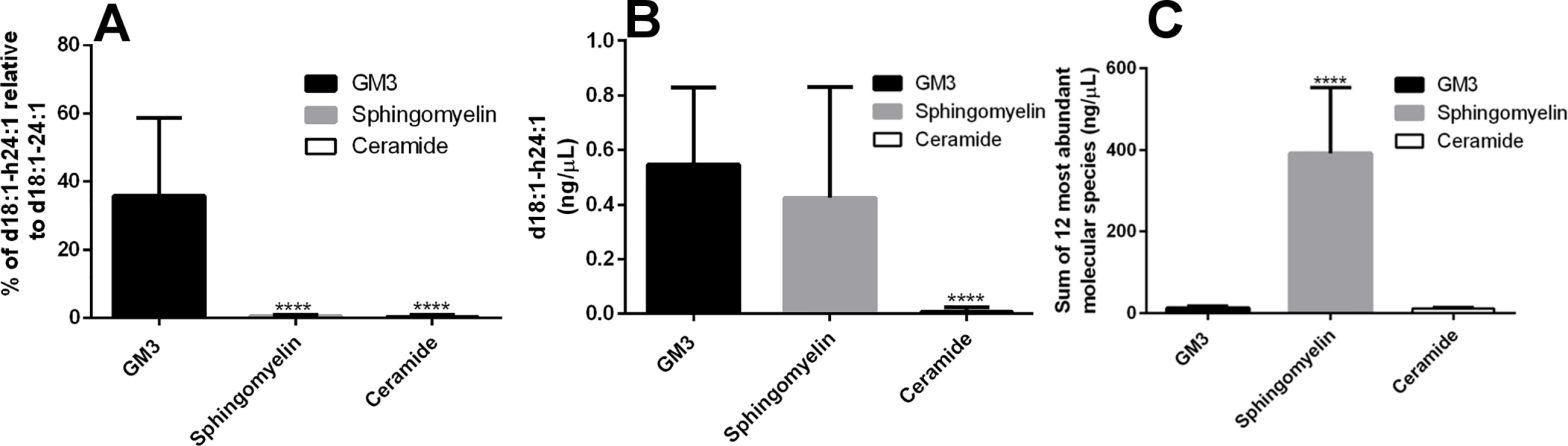
The relative abundance of d18:1-h24:1 molecular species. Percent of d18:1-h24:1 relative to d18:1–24:1 in GM3, sphingomyelin and ceramide in individuals with metabolic disease (A). The average amounts of d18:1-h24:1 (B) and the sum of the 12 most abundant molecular species (C) of GM3, sphingomyelin and ceramide in six microliters of human serum from individuals with metabolic disease. Data are reported as means ± SD. **** *P* ≤ 0.0001 sphingomyelin and ceramide vs. GM3; Mann-Whitney unpaired test.

In individuals of VFA with metabolic disease, the ratio of GM3(d18:1-h24:1) to GM3(d18:1–24:1) was significantly greater than that of sphingomyelin and ceramide. GM3(d18:1-h24:1) existed at 35.8 ± 22.9% of GM3(d18:1–24:0), whereas sphingomyelin(d18:1-h24:1) and ceramide(d18:1-h24:1) presented at 0.6 ± 0.3% and 0.4 ± 0.5%, respectively (Fig 6A). The total amount of GM3(d18:1-h24:1) in serum was also found to be greater than the same species in sphingomyelin and ceramide, measuring 0.546 ± 0.283 ng/μL compared to 0.426 ± 0.406 ng/μL and 0.011 ± 0.013 ng/μL, respectively (Fig 6B). To put the relative amounts of d18:1-h24:1 molecular species into perspective, the sums of the 12 most abundant GM3, sphingomyelin and ceramide molecular species were calculated. The amounts were 12.697 ± 4.713 ng/μL, 391.538 ± 161.703 ng/μL and 11.135 ± 3.281 ng/μL for GM3, sphingomyelin and ceramide, respectively (Fig 6C).

## Discussion

The pathogenesis of obesity induced type 2 diabetes and CVD has been reported to correspond with increased plasma sphingolipids such as ceramide and sphingomyelin [20]. Previous studies have also found human serum GM3 concentrations to be increased in type 2 diabetes accompanied by severe visceral fat accumulation [12]. In addition to increased GM3 levels a positive correlation with LDL-c in serum of patients with type 2 diabetes (r_s_ = 0.403, p = 0.012) has been observed [12]. Serum GM3 levels were also found to be elevated in patients with high levels (> 10 mg/dL) of small dense LDL, a form of LDL that is known to be especially atherogenic [21–23], a finding significant to our discovery that GM3 molecular species are correlated with CVD risk factors. Because GM3 exists as a prominent ganglioside in blood and has a negative influence on insulin signaling [6,17,24], this information is important. However, GM3 is not a single entity but rather a number of molecular species with varying ceramide moieties, each with unique biophysical properties and, presumably, physiological functions. With the fact in mind that total GM3 is elevated in metabolic syndrome we set out to explicitly determine which GM3 molecular species are implicated in metabolic disease.　

Our results confirmed the finding by Sato, et al. [12] that individuals of VFA with hyperglycemia, and even more so in individuals of VFA with metabolic disease, have increased total serum GM3 (Fig 2A), however variation within groups resulted in an alpha level greater than 0.05. Further, we also confirmed that total GM3 levels are correlated with LDL-c (Fig 4F), a finding in line with previous reports that lipoprotein associated gangliosides are increased in hyperlipidemic patients [25] and that approximately 98% of human serum gangliosides are transported by lipoproteins [26].

Upon comparing total GM3 (Fig 4) and GM3(d18:1-h24:1) (Fig 3), the advantage to monitoring individual GM3 molecular species over total GM3 becomes clear. While both total GM3 and GM3(d18:1-h24:1) are correlated with total cholesterol, LDL-c and fasting blood glucose (Figs 4E, 3E, 4F, 3F, 4A and 3A, respectively), only GM3(d18:1-h24:1) is significantly correlated with autotaxin (Fig 3G), insulin (Fig 3B) and HOMA-IR (Fig 3C); adipose-derived autotaxin contributes to adipose tissue expansion and insulin resistance in diet-induced obesity [27]. Additionally, GM3(d18:1-h24:1) is more strongly correlated with both mean IMT (total GM3 r_s_ = 0.2242 (Fig 4H) vs GM3(d18:1-h24:1) r_s_ = 0.3709 (Fig 3H)), a risk factor for atherosclerotic disease, and the diabetic indicator HbA1c (total GM3 r_s_ = 0.1820 (Fig 4D) vs GM3(d18:1-h24:1) r_s_ = 0.2803 (Fig 3D)).

It is important to note that all GM3 molecular species are not always correlated with metabolic disease risk factors (Fig 4B, 4C and 4G). Because of this, simply measuring total GM3 values to infer information about a patient’s propensity for type 2 diabetes or CVD may be misleading and result in a false positive or a missed diagnosis, *e*.*g*. in the event that elevated total GM3 levels are due to molecular species not associated with risk factors or the situation where GM3 levels are within the normal range but high levels of the risk factor associated hydroxylated species are present. Moving forward it will be essential to target specific GM3 molecular species, namely hydroxylated molecular species, to provide the most accurate and reliable biomarker information to patients and health care providers.

The molecular species containing ceramide(d18:1-h24:1) is present in GM3, sphingomyelin and ceramide and relative abundances are variable. However, the absolute and relative abundance of GM3(d18:1-h24:1) makes it the most attractive target for metabolic screening, *e*.*g*. the relative abundance of GM3(d18:1-h24:1) to GM3(d18:1–24:0) 35.8 ± 22.9%, that of sphingomyelin and ceramide 0.6 ± 0.3% and 0.4 ± 0.5%, respectively (Fig 6A) and the absolute amount of each GM3(d18:1-h24:1), sphingomyelin(d18:1-h24:1) and ceramide(d18:1-h24:1) in human serum is 0.546± 0.283 ng/μL, 0.426 ± 0.406 ng/μL and 0.011 ± 0.013 ng/μL, respectively (Fig 6B). The finding that the molecular species GM3(d18:1-h24:1) is most abundant is quite remarkable, considering the degree that total serum sphingomyelin outweighs total serum GM3. For example, the 12 most abundant sphingomyelin molecular species total 391.538 ± 161.703 ng/μL, over thirty times more than the same measure of GM3 which is 12.697 ± 4.713 ng/μL (Fig 6C). This underscores the significant and specific upregulation of GM3(d18:1-h24:1) in individuals with risk factors for type 2 diabetes and CVD.

The formation of hydroxylated sphingolipids begins with fatty acid 2-hydroxylase (FA2H) converting acyl-CoAs to 2-hydroxylated-CoAs and 2-hydroxylted-CoAs are transferred to dihydrosphingosine by an isoform of ceramide synthase, of which there are six capable. Des-1 desaturase then converts hydroxy fatty acid (hFA)-dihydroceramide to hFA-ceramide which acts as a precursor for complex hFA-sphingolipids [28]. Another possible means for the formation of hydroxylated sphingolipids, that is FA2H independent, involves the enzyme phytanoyl-CoA-2-hydroxylase (PHYH) of the α-oxidation pathway. In this scenario hFA would be synthesized in peroxisomes and transported to the endoplasmic reticulum (ER) where hFA-ceramide is formed. Or, alternatively, PHYH would be trafficked to the ER to participate in the synthesis of hFA-ceramide [28]. A third possibility that does not involve hFA-ceramide serving as the precursor for hFA-sphingolipid synthesis is that of direct hydroxylation of non-hydroxy fatty acid-containing sphingolipids, a possibility supported by studies examining the formation of hFA-sphingolipids in tetrahymena [29]. Although we presently do not have enough evidence to indicate the means by which hydroxylated GM3 molecular species are enriched in the individuals of VFA with metabolic disease, the observation that hydroxylated GM3 molecular species are far more abundant than hydroxylated ceramide molecular species seems to point to the mechanism of direct hydroxylation. It is interesting to note that a linear correlation has been reported between GM3 molecular species containing hydroxylated acyl chains and aging in the human liver [30], the site of excretion of gangliosides into mammalian sera [25,31].

The observation that GM3 treatment of 3T3-L1 adipocytes induces a fourfold increase in IL-6, PAI-1 and TNF-α mRNA suggests that gene expression of proteins involved in obesity induced thrombosis and inflammation may be dependent on its abundance [20]. Moving forward we will explore the idea that a specific increase in hydroxylated GM3 molecular species (Fig 2B), particularly GM3(d18:1-h24:1) (Fig 1A), may influence the regulation of membrane-associated signal transduction events. An idea that seems plausible in light of the finding that hFA derivatives have been shown to regulate microdomain structure by modulating the composition of ordered microdomain components [32] and membrane lateral diffusion [33]. Future investigations will aim to elucidate potential underlying mechanisms linking hydroxylated GM3 molecular species with the progression of insulin resistance and atherosclerosis. Planned experiments involve examining the impact that synthetic hydroxylated GM3 molecular species have on signaling events associated with IR activation. Additionally, owing to the fact that the GM3 precursor ceramide has been reported to be positively correlated with obesity and metabolic disease [34], to obtain a more global view of sphingolipid metabolism, it will be important to analyze full profiles covering ceramide, glucosylceramide, lactosylceramide and sphingomyelin molecular species for these patients in the future.

## Supporting Information

S1 Table Mass spectrometer settings and MRM transition pairs. (PDF)
