# Supplementary material for: Identification of Ganglioside GM3 Molecular Species in Human Serum Associated with Risk Factors of Metabolic Syndrome
Source: PLoS One. 2015 Jun 23;10(6):e0129645. doi: 10.1371/journal.pone.0129645 (PMC4477979; doi:10.1371/journal.pone.0129645)
Supplement: S1 Table — (PDF) [file pone.0129645.s001.pdf]

S1 Table. Mass spectrometer settings and MRM transition pairs.

	Molecular species	Q1 *m/z*	Q3 *m/z*	CE (eV)	S-lens RF amplitude
Ceramide					
$[M-H]^-$	d18:1-17:0	550.5	263.2	29	204
	d18:1-24:1	646.6	263.2	29	204
	d18:1-h24:1	662.6	263.2	29	204
Sphingomyelin					
$[M-H+Ac]^-$	d18:1-17:0	775.5	167.9	46	171
	d18:1-24:1	871.6	167.9	46	171
	d18:1-h24:1	887.7	167.9	46	171
GM3					
$[M-H]^-$	d18:1-16:1	1149.7	289.9	53	276
	d18:1-16:0	1151.7	289.9	53	276
	d18:1-h16:1	1165.7	289.9	53	276
	d18:1-[^{13}C]16:0	1167.9	289.9	53	276
	d18:1-18:1	1177.7	289.9	53	276
	d18:1-18:0	1179.7	289.9	53	276
	d18:1-h18:1	1193.7	289.9	53	276
	d18:1-h18:0	1195.7	289.9	53	276
	d18:1-20:1	1205.7	289.9	53	276
	d18:1-20:0	1207.7	289.9	53	276
	d18:1-21:1	1219.7	289.9	53	276
	d18:1-21:0	1221.7	289.9	53	276
	d18:1-h20:0	1223.7	289.9	53	276
	d18:1-22:1	1233.7	289.9	53	276
	d18:1-22:0	1235.7	289.9	53	276
	d18:1-h21:0	1237.7	289.9	53	276
	d18:1-23:1	1247.7	289.9	53	276
	d18:1-23:0	1249.7	289.9	53	276
	d18:1-h22:0	1251.7	289.9	53	276
	d18:1-24:1	1261.8	289.9	53	276
	d18:1-24:0	1263.8	289.9	53	276
	d18:1-h23:0	1265.8	289.9	53	276
	d18:1-h24:1	1277.8	289.9	53	276
	d18:1-h24:0	1279.8	289.9	53	276

Q, quadrupole; CE, collision energy; S-lens, stacked-ring ion guide; RF, radio frequency.
